# Online Learning Algorithm for Time Series Forecasting Suitable for Low Cost Wireless Sensor Networks Nodes

**DOI:** 10.3390/s150409277

**Published:** 2015-04-21

**Authors:** Juan Pardo, Francisco Zamora-Martínez, Paloma Botella-Rocamora

**Affiliations:** ESAI—Embedded Systems and Artificial Intelligence Group, Escuela Superior de Enseñanzas Técnicas, Universidad CEU Cardenal Herrera, C/San Bartolomé, 46115 Valencia, Spain; E-Mails: francisco.zamora@uch.ceu.es (F.Z.-M.); pbotella@uch.ceu.es (P.B.-R.)

**Keywords:** wireless sensor networks, artificial neural networks, on-line Back-Propagation, ambient intelligence, energy efficiency

## Abstract

Time series forecasting is an important predictive methodology which can be applied to a wide range of problems. Particularly, forecasting the indoor temperature permits an improved utilization of the HVAC (Heating, Ventilating and Air Conditioning) systems in a home and thus a better energy efficiency. With such purpose the paper describes how to implement an Artificial Neural Network (ANN) algorithm in a low cost system-on-chip to develop an autonomous intelligent wireless sensor network. The present paper uses a Wireless Sensor Networks (WSN) to monitor and forecast the indoor temperature in a smart home, based on low resources and cost microcontroller technology as the 8051MCU. An on-line learning approach, based on Back-Propagation (BP) algorithm for ANNs, has been developed for real-time time series learning. It performs the model training with every new data that arrive to the system, without saving enormous quantities of data to create a historical database as usual, *i.e.*, without previous knowledge. Consequently to validate the approach a simulation study through a Bayesian baseline model have been tested in order to compare with a database of a real application aiming to see the performance and accuracy. The core of the paper is a new algorithm, based on the BP one, which has been described in detail, and the challenge was how to implement a computational demanding algorithm in a simple architecture with very few hardware resources.

## 1. Introduction

Wireless Sensor Networks (WSNs) have been widely considered as one of the most promising present and future technologies. In fact, the latest advances in wireless communication technologies have made it possible to develop tiny, cheap and smart sensors embedded in a small physical area, with wireless network capabilities, that provides huge opportunities for a vast variety of applications. Some common examples can be found, such as industrial monitoring processes, machine health monitoring, physical and environmental conditions monitoring, *etc.* [1]. However, one of its most promising applications is on smart homes and ambient intelligence, which makes it feasible to provide scalable intelligent networks of sensors/actuators according to new home technologies appear on the market. WSNs can be used to provide more convenient and intelligent living environments for human beings and can be embedded into a house to develop an autonomous home network. The present paper uses a WSN to monitor and forecast the indoor temperature in a smart home, based on low resources and low cost microcontroller technology.

Several studies say that in the European Union about 40% of total primary energy demand corresponds to buildings' consumption [2]. At home, more than a half of such consumption is produced by HVAC (Heating, Ventilating and Air Conditioning) systems [3]. The indoor temperature is the most crucial variable that determines the utilization of such systems and thus has a major effect on the overall energy expenditure. For that reason, it is still necessary to develop new intelligent systems at home to manage the demand of energy efficiently, considering a plausible balance between consumption and comfort. To develop such intelligent systems, artificial intelligence techniques, as forecasting, can be applied. Soft computing has been widely used in real-life applications [4,5]. Furthermore, the estimation of Artificial Neural Network (ANN) models by using machine learning techniques have been applied for a wide range of applications, and are also devoted to developing energy systems [2,6–8]. The problem is that such techniques normally require high computational resources and historical data, and the traditional training method is based on batch learning, as for example Back-Propagation (BP) algorithm and its variants. But for most applications, it could consume from several minutes to some hours and further the learning parameters must be properly chosen to ensure the convergence (*i.e.*, learning rate, number of learning epochs, stopping criteria, *etc.*). In a batch learning system, when new data are received then it is performed a retraining jointly with its past data, thus consuming a lot of time as it is mentioned in [7,9].

Nevertheless, as an alternative, an on-line learning approach could perform the model training with all new incoming data. In fact, when it is necessary to learn a model from scratch or to adapt a pre-trained one in a totally unknown scenario, on-line learning algorithms can be applied successfully [10]. Thus, we talk with regard to Stochastic Gradient Descent Back-Propagation (SGBP) as a particular variant of BP for sequential or on-line learning applications. Through sequential or on-line learning methods, the training observations are sequentially presented to the learning algorithm. Therefore, when new data arrive at any time, they are observed and learned by the system. In addition, as soon as the learning procedure is completed the observations are discarded, without having the necessity to store too much historical information, that also implies less necessity of additional physical storage. In conclusion, the learning algorithm has no prior knowledge about how many training observations will be presented, although it is possible to produce a better generalization performance at a very fast learning speed [9] and needs less computing resources that accomplish with our idea of integrating this technology in a low cost embedded system inside a WSN framework.

The present research group is concerned with regarding the idea of being able to design new intelligent systems, with few hardware resources, to predict values of strategic variables related to energy consumption, *i.e.*, low cost and small predictive systems. For that purpose, sequential learning algorithms have demonstrated their feasibility to achieve such objectives. This also implies having cheap hardware devices embedding complex artificial intelligence techniques for forecasting in unknown environments, but also with affordable computing and economical costs. As far as we know, it is usual to employ WSN as the monitoring system that feeds an ANN implemented in a personal computer, as an ANN requires some complex calculations and that also means using wide data storage. However, what it is proposed in this paper is whether or not it is feasible to implement, inside a node of a WSN, an ANN that performs predictions with an acceptable resolution in its estimations. Consequently, in this paper, we present a preliminary model able to generate low error predictions over short periods of learning time. Regarding the innovation of the paper, it has been developed a new on-line learning algorithm, based on a BP framework, which is able to preprocess real-time continuous input data, incoming in a non-deterministic way, from a wireless environment, being also feasible to be implemented in devices with very low hardware resources, *i.e.*, with important hardware constraints.

The paper is organized as follows, in Section 2, we describe the framework in which the present work have been developed, *i.e.*, a wireless sensor network, describing the hardware of the different nodes and the network topology in order to slightly describe the experimental setup. In Section 3, we explain the approach that has been followed to forecast time series using an on-line learning paradigm based on Back-Propagation (BP) algorithm for Artificial Neural Networks. Section 4 depicts in detail the algorithm developed to be implemented in a low resources microcontroller as the 8051MCU. Finally, Sections 5 and 6 the experimental results and the discussion and conclusions explain the present research and draw some future ideas to continue the present project.

## 2. Wireless Sensor Network Architecture

Basically, a WSN consists of a large number of low-cost, low-power and multifunctional sensor nodes that are deployed in an environment devoted for monitoring tasks, but also for controlling as current networks are bidirectional, where sensor activity can be controlled. Actually, such sensor nodes, small in size, are equipped with sensors, embedded microprocessors and radio transceivers, and therefore they have also capabilities for data processing and communication over short distances, via a wireless medium, in order to collaborate to accomplish a common task [1].

A WSN is built of nodes which can vary in number from a few to several hundred or even thousands, in which each node is connected to one or several sensors. Each sensor network node is typically divided into several parts: a radio transceiver with an internal antenna or connection to an external one, a microcontroller, an electronic circuit for interfacing with the sensors and an energy source, usually a battery or an embedded device for energy harvesting [11]. Furthermore, the cost of sensor nodes is also variable, ranging from a few to hundreds of Euros, depending on the complexity of the individual sensor nodes. Additionally, size and cost constraints on sensor nodes also result in the corresponding constraints on resources such as energy, memory, computational speed and communications bandwidth. Finally, about the topology of the WSNs, this can vary from a simple star network to an advanced multi-hop wireless mesh network. In addition, the propagation technique between the hops of the network can be routing or flooding [12,13].

Besides sensor networks have the following unique characteristics and constraints, as it is stated in [1]:

- Dense Node Deployment. The number of sensor nodes can be of several orders of magnitude.
- Battery-Powered Sensor Nodes. Being in some situations difficult or even impossible to change or recharge their batteries.
- Severe Energy, Computation, and Storage Constraints. Sensor nodes are resource limited. This work is focused on this constraint.
- Self-Configurable. Sensor nodes configure themselves into a communication network.
- Application Specific. A network is usually designed and deployed for a specific application.
- Unreliable Sensor Nodes. They are prone to physical damages or failures.
- Frequent Topology Change. Network topology changes due to node failure, damage, addition, energy depletion, or channel fading.
- No Global Identification. It is usually not possible to build a global addressing scheme for a sensor network because it would introduce a high overhead for the identification maintenance.
- Many-to-One Traffic Pattern. In most sensor network applications, the data sensed by sensor nodes flow from multiple source sensor nodes to a particular sink.
- Data Redundancy. The data sensed typically have a certain level of correlation or redundancy.

Furthermore, the characteristics of sensor networks and requirements of different applications have a decisive impact on the network design objectives in terms of network capabilities and network performance. Thus, typically influential design objectives for sensor networks include the following several aspects: small node size, low node cost, low power consumption, self-configurability, scalability, adaptability, reliability, fault tolerance, security, channel utilization and QoS support [1].

Moreover, a typical scheme of a wireless sensor network is composed of a set of nodes that transmits the information acquired to a sink node. This one is usually devoted to collecting and centralizing all the information that comes from the network to a Personal Computer (PC), in order to store big quantities of data in a persistent device. Thus, the information collected can be treated for on-line or later analysis. But for the purposes of our study, we don't want to dump the information acquired by the network to a PC. It is desired to use such information, as it will be described later, to train a neural network, implemented inside a sink node, trying to develop an autonomous forecasting system.

**Figure 1:**
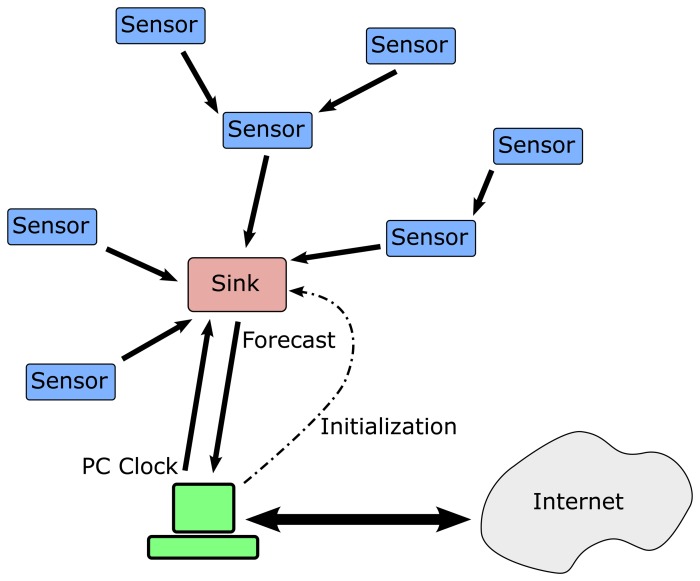
Wireless sensor network scheme.

Figure 1 shows the wireless sensor network scheme designed in the present study. The network is composed of five nodes, but more nodes can be added in the way the figure displays. There is a sink node connected to a PC for configuration and validation purposes and four sensor nodes that capture the temperature inside a room. Sensor nodes can work as repeaters allowing low power transmit modes in order to extend battery life.

### 2.1. Nodes Description

As mentioned previously, our wireless sensor network consists of two kinds of nodes, four sensor nodes and one sink node. Both are based on the same technology, although, of course, in charge of different tasks. All nodes are based on the CC1110F32 microcontroller (Texas Instruments, Dallas, TX, USA) [14]. The CC1110F32, is a true low-power sub-1 GHz system-on-chip (SoC) designed for low power wireless applications. It combines the excellent performance of the state-of-the-art RF transceiver CC1101 with an industry-standard enhanced 8051MCU, up to 32 KB of in-system programmable flash memory and up to 4 KB of Random Access Memory (RAM), and many other powerful features. The radio frequency range can be chosen from: 300–348 MHz, 391–464 MHz and 782–928 MHz. Its small 6 × 6 mm package makes it very suited for applications with size limitations. The CC1110F32 is highly suited for systems where very low power consumption is required. This is ensured by several advanced low-power operating modes. Additionally, its low power demands (16 mA for transmission at 10 mW and 18 mA for reception) make it suitable for battery-powered systems.

**Figure 2:**
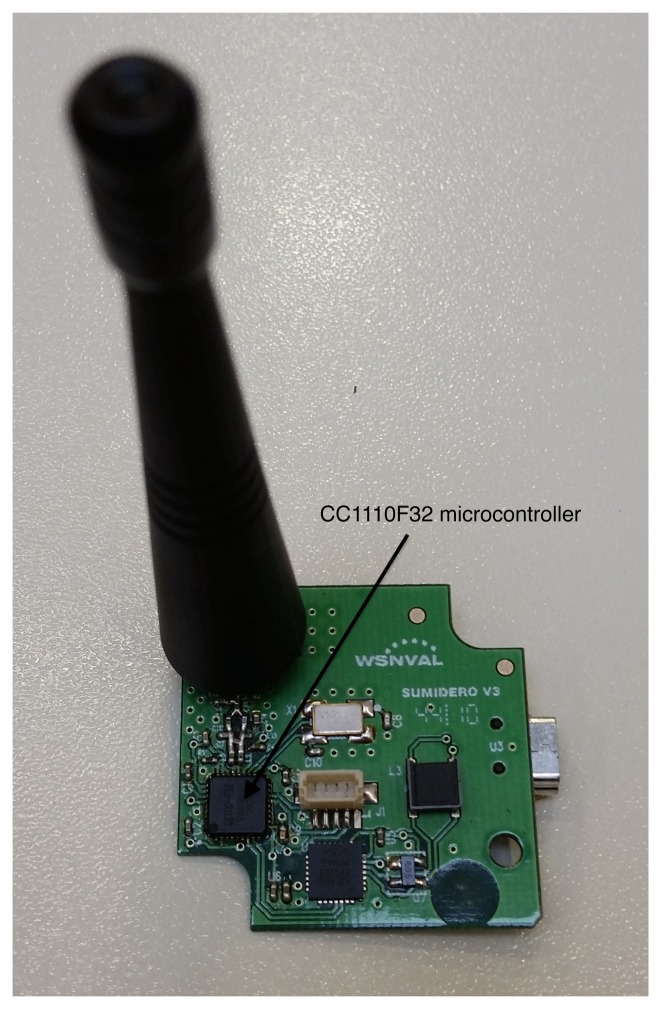
Sink node.

**Figure 3:**
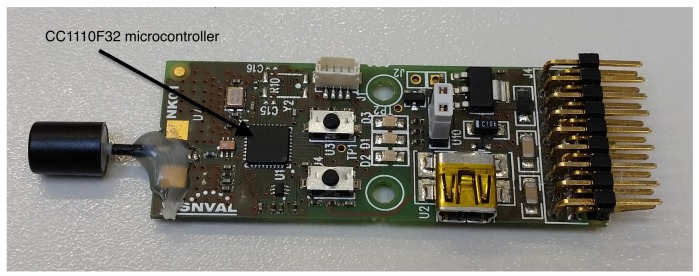
Sensor node.

**Sink node** (Figure 2): Receives all the wireless information transmitted by the sensor nodes. It is switchable to a PC through a USB connection for configuration purposes, and it works at a speed of 868 MHz. The USB is also its power connection, its dimensions are 40 × 40 × 90 mm and it is able to work within a temperature range of −40 to 85 °C. 2-FSK, GFSK, MSK, ASK, and OOK modulation formats are supported and includes a 128-bit AES security coprocessor. Its power transmission is 10 mW and high sensitivity of −110 dBm at 1.2 kBaud. It has an external exchangeable antenna and programmable data rate up to 500 kBaud. The processor also includes one timer of 16 bits and three timers of 8 bits and on-chip hardware debugging.
**Sensor node** (Figure 3): There are four sensor nodes, distributed in a room for the present study, that constantly send the temperature they pick up from the ambient. All four have been calibrated with a digital thermometer at the same point and later have been distributed with similar distances to the sink node. The sensor node is based on the same SoC as the sink node and additionally it has a temperature sensor, three color leds, two buttons (one reset and another for general purpose) and an input/output expansion connector to install other sensor/actuators. Its power supply can be through batteries, USB or electric power. In addition, its antenna, which is integrated in the circuit, can reach up to 290 m without repeaters. It is possible to connect more sensor nodes, as much as are needed, depending on the application target. They autonomously connect themselves to the network and start to send temperatures immediately.

The hardware of the sensor and sink nodes have been developed by the company Wireless Sensor Networks Valencia SL for applications of monitoring and controlling of industrial systems, domotics, security and alarm systems, sensorization, telemetry, *etc.* In this study, such nodes have been programmed to deploy the wireless sensor network and to implement an ANN for on-line learning purposes.

There are other research works developed with more advanced architectures, as with ARM microcontrollers. Nevertheless, it is true that the Texas Instruments CC1110F32 MCU is highly accepted in a lot of areas, and a leader in the market as far as we know, for Industrial monitoring and control applications. Moreover, it is also a *true low power SoC*, excellent for WSNs. If the application is to massively deploy such devices, they are cost competitive and really small. These devices accomplish with the simplest architecture that makes feasible the implementation of our algorithm. The SoC has been signaled in the pictures to see the size.

## 3. Time Series and Forecasting

In smart homes is common to measure and monitor environmental variables related to comfort and energy consumption. This kind of data are recorded over a period of time (every second, every minute…). Studying the behavior of these variables and forecasting their values in the future allows to manage energy resources more efficiently.

A collection of data recorded repeatedly over the time represents a time series from the point of view of an statistical treatment of data. Thus, a time series is a collection of data recorded over a period of time from any interesting process. They can be formalized as a sequence of scalars from a variable *x* obtained as output of the observed process:

(1) $$x_{t_{1}},\ldots ,x_{t_{i-1}},x_{t_{i}},x_{t_{i+1}},\ldots$$

There are several methods that are appropriate to model a time series. Time series data have an internal structure, such as auto-correlation, trend or seasonal variation and these features should be considered by the modeling and forecasting methods that can be used.

Several approaches has been widely employed, such as smoothing and curve fitting techniques and autoregressive and moving average models [15–17]. However, the idea of restricting the storage and computational structure, of our present study, don't allow us using them. Other methods framed in regression models have been implemented to carry out the main purpose of this paper.

In order to simplify as much as possible the forecasting process, in this work, two different low resource implementable models have been proposed: a linear model (that is, a Perceptron) and a Multilayer Perceptron (MLP) model with one hidden layer. Both methods have been implemented and introduced into the sink node.

Furthermore, with the purpose of comparison and in order to know the behavior of these two algorithms, a baseline standard method has been developed in a PC. A linear model (a perceptron) has been selected and estimated by a standard method: Bayesian estimation. A Baseline model approach will represent the results of a standard model, estimated with more storage prerequisites and with more complex computational necessities. It is expected that the results of this baseline model will be better than those provided by the constrained models. However, this comparison will allow to assess, based on the magnitude of errors obtained, the feasibility of using the algorithms implemented in the device which has been proposed in this paper.

### 3.1. Measurements of Average Model-Performance

Measurements of average error or model performance are based on statistical summaries of the differences between each target value vector y[*t*] and its predicted vector ŷ[*t*]. In general, the most used measure of average model-performance is MAE (Mean Absolute Error):

(2) $$\text{MAE}\left(t=i\right)=\frac{1}{q}\sum_{z=1}^{q}\left|{\hat{y}}_{z}\left[t\right]-y_{z}\left[t\right]\right|$$

The MAE averaged over several time instants in a data set 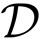 will be denoted as MAE^★^:

(3) $$MAE^{*}=\sum_{i}\frac{\text{MAE}\left(i\right)}{\left|D\right|}$$

being | 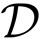
| the number of samples in 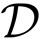.

### 3.2. On-Line Learning in Time Series Forecasting

An on-line learning approach, as the proposed in this work, is a class of sequential learning paradigm where data frames income on real-time. The present work deals with data frames that are not equidistant in time. This enables the WSN to be modified at any time, and it allows that the failure of any node at any moment was not a critical issue. In Section 4 we explain how the algorithm preprocesses the raw incoming data frames to compute equidistant and first-order differentiated time series values. Furthermore, the proposal of having a low hardware resources constraint for the algorithm implementation, only lets us to store a short buffer of incoming data, forcing the time series forecasting model to update its parameters with every new value that arrives.

As far as we know, the convergence and behavior of on-line algorithms has been studied, by machine learning scientists, as well as the well known Stochastic Gradient Descent and its variants, and particularly the error Back-Propagation (BP) algorithm for Artificial Neural Networks [18]. The on-line BP is stochastic when the training samples are learned in a random order, probably with replacement. This randomized update process yields a noisy gradient computation problem, which is usually overcome by a higher number of model updates, compared with the batch version of the same BP algorithm. Therefore, in the standard framework for on-line BP, random traversal of the dataset yields better results.

However, this work is focused on a straightforward algorithm for real-time time series learning, that follows an on-line BP strategy but in a sequential way. Such a problem statement has the advantage of its possible implementation for low resources devices, but it lacks of the random data traversal of usual on-line BP approaches, which could be a source of problems in the convergence of the algorithm. As expected, this problem could be more shocking as more complex the model is.

A possible refitting, out of the purpose of this work, could be the implementation of a random skip procedure, that allows for ignoring incoming values depending on a probability parameter. Such a skip procedure introduces a trade-off between the stochastic behavior in gradient computation and the number of samples used to train the model. Additionally, other interesting extensions are possible for more powerful devices, as a short buffer of input/output samples, selected randomly from the data stream (known as reservoir sampling), that performs a model update every *k* random steps. Even more complicated algorithms are also possible, where the skip decision rule depends on the behavior of the model with current data samples.

Different variations of on-line BP algorithm can be taken into consideration for real-time fitting. It is true that Least Mean Squares training have problems when input variables are in different scales. Thus, Normalized Least Mean Squares [19] training is proposed to tackle with such scale problems. However the developed system adopt first-order differences of input data and all input variables belong to the same signal, that is, all input variables have the same scale. Other issue of the on-line training algorithm, when exists capture error, is the not convergence to a unique value, but to a minimum capture error. This question could be solved by incorporating dead-zones in the algorithm as stated in [19].

### 3.3. Baseline Method: Standard Bayesian Linear Model Estimation

In linear regression models, observations consist of a response variable in a vector **y** and one or more predictor variables in a matrix **X**. The response vector has *n* elements, concerning to *n* observations, so the matrix **X** will have *n* rows and *p* columns corresponding to the number of predictors or covariates (*n* ≥ *p* + 1 for non-denerate variance parameter estimation). Additionally, it is usual to introduce an intercept parameter in the model, thus a column of **X** is a column completed by number ones. In the same way, other exogenous variables, which could condition the forecast of the response variable, could be introduced as additional columns in matrix **X**. Consequently, the parameters are the regression coefficients, **w**, and the error variance of the fitted model, *e*^2^. Thus, the linear model could be written in its matricial form:

(4) $$\text{y}|\text{w},e^{2},\text{X}∼N\left(\text{Xw},e^{2}{\text{I}}_{n}\right)$$

where **I***_n_* represents the *n* × *n* identity matrix.

Occasionally, more than one future value must be predicted, hence the model receives at each moment as input the *p* last values in time series and must predict next *q* values, thus **y** and **w** becomes in a *n* × *q* and *p* × *q* matrices **Y** and **W**. In this framework, at time *t* it is available one new element of *p* past values in the time series ([*x_t_*_−_*_p_*_+__1_,…, *x_t_*_−1_, *x_t_*]) that was considered as covariates in the simple linear model to forecast *q* further values (*x_t_*_+1_,…, *x_t_*_+_*_q_*) = (*y*_1_,…, *y_q_*). In consequence, it has been built a simple linear model for each prediction:

(5) $$\begin{array}{cc} {\text{Y}}_{.i}|{\text{W}}_{.i},e_{i}^{2},\text{X}∼N\left({\text{XW}}_{.i},e_{i}^{2}\text{I}\right) & i=1,\ldots ,q \end{array}$$

where **M**.*_i_* denotes *i*-column at any **M** matrix.

This forecasting process needs *n* + 1 observations to start to generate predictions (**X** represents a (*n* + 1) × *p* matrix and **Y** a (*n* + 1) × *q* matrix) and *q* linear models must be estimated (one for each prediction) in each time step.

Some assumptions must be considered about the classical linear model, such as **X** should be full rank (no collinearity among predictors), exogenous predictors and not auto-correlated errors. Using this sort of models to represent time series, that uses lagged predictors to incorporate feedback over the time, means that some of these assumptions are violated. Autoregressive processes comparable to this one introduce violations of classical linear model assumptions that lead to biased parameters estimations. Nevertheless, it could be improved, considering more complex covariance structures. As it has been mentioned before, a random skip procedure could solve some of these problems. However, in this case, the primary goal of our work has been focused on building an on-line estimation model process. Moreover, it has to be able to perform predictions with an acceptable resolution in its estimation, and with low computing and storage resources.

A Bayesian framework [20] provide a natural way to perform an on-line estimation. Such methods make it possible to incorporate scientific hypothesis or prior information based on previous data, by means of the prior distribution. Nevertheless, in the absence of prior information, a Bayesian estimation of the parameters is made in an objective context, with objective prior distributions for them, that are estimated with information provided by the data. In addition, when prior information is available or some scientific hypothesis have been assumed, a Bayesian estimation could be made in a subjective framework, incorporating prior information into parameter prior distributions.

In the context of this work, the first step is a Bayesian parameter estimation, made by means of a non-informative prior distribution for the parameters. When the first parameter estimation is available then the estimated model is used to generate the predictions. The predictive distribution, **Ŷ**, given a new set of predictors **X***_p_* has mean:

(6) $$\begin{array}{cc} {\hat{Y}}_{.i}={\text{X}}_{p}\hat{W}{\left[0\right]}_{.i} & i=1,\ldots ,q \end{array}$$

where **Ŵ**[0] is calculated with first *n* + 1 data elements available **X** and **Y** as:

(7) $$\hat{\text{W}}{\left[0\right]}_{.i}={\left({\text{X}}^{\text{⊤}}\text{X}\right)}^{-1}{\text{X}}^{\text{⊤}}{\text{Y}}_{.i}$$

Therefore, it demonstrates that it is necessary to solve matrix products and inverse matrices with dimension (*p* × *p*). Inversion matrices could be avoided employing, for example, *QR* decomposition in **X** matrix, but in terms of computational and storage cost remains as an expensive process.

After this first step, the system has prior information available, that must be incorporated in a future parameters estimation. The way to introduce previous information, with new data to improve such parameters estimation, is by using informative prior distribution. In the context of this work, linear regression with an informative prior distribution was used. At time *t*, previous estimation on parameter assessment was employed in prior distribution of parameters and new data was utilized to re-estimate model parameters. Furthermore, if at time *t* the last parameter estimation is **Ŵ**[t − 1] and we have new data **X**[*t*] and **Y**[*t*], the new appraisal at this point of time can be computed by treating the prior as additional data points, and then weighting their contribution to the new estimation [20]. To perform the computations, for each prediction value **Y**[*t*]_._*_i_* (*i* = 1, …, *q*) it is necessary to construct a new vector of observations ${\text{Y}}_{.i}^{*}$ with new data and last parameters assessment, and predictor matrix X*, and weight matrix **Σ** based on previous variance parameters estimation as follows (more details can be read at the Appendix):

(8) $${\text{Y}}_{.i}^{*}=\left[\begin{array}{c} \text{Y}{\left[t\right]}_{.i}\\ \hat{\text{W}}{\left[t-1\right]}_{.i} \end{array}\right]$$

(9) $${\text{X}}^{*}=\left[\begin{array}{c} \text{X}\left[t\right]\\ {\text{I}}_{p} \end{array}\right]$$

where **I***_p_* represents a *p* × *p* identity matrix. The new parameters appraisal at time t could be written as:

(10) $$\begin{array}{cc} \hat{\text{W}}{\left[t\right]}_{.i}={\left({\text{X}}^{*\text{⊤}}\Sigma^{-1}{\text{X}}^{*}\right)}^{-1}{\text{X}}^{*\text{⊤}}\Sigma^{-1}{\text{Y}}_{.i}^{*} & i=1,\ldots ,q \end{array}$$

Thus, the predictive distribution, **Ŷ**, given a new set of predictors **X***_p_* has as a mean:

(11) $$\begin{array}{cc} {\hat{\text{Y}}}_{.i}={\text{X}}_{p}\hat{\text{W}}{\left[t\right]}_{.i} & i=1,\ldots ,q \end{array}$$

Complexity in second and posterior steps in the assessment process is higher than the first step. The number of matrix inversions and computational complexity has increased now.

Consequently, assuming a simple linear model, with the limitations mentioned before that this type of models have to model dynamic processes, it represents a computational and storage costs too high to be implemented in a device with low hardware resources, as we are concerned in this paper. The Bayesian standard linear model parameters estimation is a simple process but with high computational resource requirements. Thus, it is necessary to solve some inverses matrices, whose cost is too expensive in the context of this paper.

In conclusion, this model has been considered as baseline model to compare the results of both algorithms related with the two ANN models that have been implemented in the sink node.

## 4. Sequential On-Line Back-Propagation Algorithm for Devices with Low Resources

This section describes the considered implementation of an on-line version of BP algorithm [18] for devices with very few memory and computing resources, as the 8051 microcontroller included in the sink node. The BP algorithm is able to train any kind of ANN. As it was stated at Section 3.2, the present research proposes the utilization of a sequential version of on-line BP, and compares two different models with previous stated baseline: a linear model (that is, a perceptron) as shown in Equation (12) and equivalent to the model of Equation (5), and a MLP with one hidden layer as shown in Equation (13):

(12) $$\hat{\text{y}}={\text{W}}_{1}\cdot \text{x}+{\text{b}}_{1}$$

(13) $$\hat{\text{y}}={\text{W}}_{2}\cdot s\left({\text{W}}_{1}\cdot \text{x}+{\text{b}}_{1}\right)+{\text{b}}_{2}$$

being **W***_j_* weight matrices, **b***_j_* bias vectors, **x** the input and **ŷ** the output of the ANN. The Appendix B describes the derivation of BP algorithm to train any kind of ANN, from perceptrons to MLPs with any number of hidden layers. Moreover, the input vector x can also be extended with other exogenous variables that could influence the output response variable. BP is a kind of first order gradient descent algorithm, therefore it needs the computation of partial derivatives over **W***_j_* and **b***_j_*. BP algorithm has been chosen because of its simplicity, it depends in algebra operations as dot products, matrix-vector products and component-wise products. The proposed MLP has the logistic activation function in the hidden layer and the activation function can be implemented by means of an exponential one. Such operations are available by hardware and/or software libraries in 8051 microcontroller, and the memory requirements to implement these operations depend on the complexity of the ANN developed:

- In the case of the perceptron, it needs a weights matrix **W**_1_ of size *p* × *q*, a bias vector with *q* elements, input and output vectors with size *p* and *q*, and an output gradients vector with *q* elements. The BP algorithm would need *p* × *q* + *p* + *3q* real numbers in memory to work with the perceptron, lets consider *p* = *q* = 8. Thus, it would need 96 real numbers.
- In case of MLP with one hidden layer of length *h*, it needs two weights matrices **W**_1_ and **W**_2_. The first one with size *p* × *h*, the second one with *h* × *q*; two bias vector, the first one with *h* and the second one with *q* elements; the input, hidden and output vectors, with size *p*, *h* and *q* respectively; and the output and hidden gradients vector with *q* and *h* elements also correspondingly. The BP algorithm needs *p* × *h* + *h* × *q* + 2*p* + 3*h* + 2*q* real numbers, lets consider *p* = *h* = *q* = 8. Thus, it would need 184 real numbers in memory.

Using 32 bit precision for real numbers, the memory resources of BP algorithm for one hidden layer MLP when *p* = *h* = *q* = 8 are 184 × 4 = 736 bytes.

A BP algorithm has been introduced into the source code that has been implemented for the sink node. This algorithm has been split in three parts and basically computes differences in mean temperature every quarter (15 min), and handles these data to learn the ANN model. The memory requirements of this algorithm and its characteristics in detail will be described in the next sections. For the sensor nodes, the source code is not described as they only send data to the sink node, the temperature.

### 4.1. Main Loop Algorithm

The experimental setup consisted of the equidistant placement of four sensor nodes in the living room of a solar house. They send continuously the temperature to a sink node that is in charge of predicting the mean temperature for the next hours. The sink node is connected to a PC, mainly for some configurations and power reasons, and it was placed at a small work place in the living room. The sink node sends the temperature predictions to the home's central control. It is an application that was developed by the present group to monitor and control all the energy systems for the purpose of the Solar Decathlon competition [21]. Its name is CAES (Computer-Aided Energy Saving System) system [8].

The main loop procedure implemented in the sink device is shown in Algorithm 1. Before the main loop, the sink node starts with some initializations, as the board support package, the minimal radio frequency (RF) interface and the rest of issues corresponding to the wireless network. To do that, it has been compiled the SimpliciTI protocol from Texas Instruments, that is a simple low-power RF network protocol aimed at small RF networks. All of those aspects correspond to the initializeWSN() function. After that, the weights initialization, required by the ANN, are established through the function initializeWeights(). This can be done in a random way, thus the ANN starts from scratch or it can be also done reading such weights from the computer, in this way the system starts with a pre-trained ANN. It was decided to define the parameters randomly.

The core of the main loop first receives from each sensor node a data frame (*v*), which corresponds to the waitForDataFrame() procedure. It includes the temperature and some information to identify the node sender. Sensor nodes send the temperature constantly, and as they are equidistant placed in a medium size room, the difference in temperature among them is minimum, thus it can be considered redundant for the present application. Although the most important for our study is to be able to process continuous messages coming from different wireless nodes to perform an on-line learning as stated before. When a data frame is received, its time stamp (*t*) is collected through the function askForCurrentTimeStamp() and then it is called the subroutine processSampleOnLine(*v*, *t*). Such a function receives a time stamp and a temperature value, and computes averages of quarters (15 min) feeding these averages to the ANN. Such averages are computed as the integration between previous (time, temperature) pair and the current one. When a quarter is ready, it is given to the ANN as described later.

This main loop has negligible memory requirements; it only uses static variables to pass the data between the different subroutines. The memory consumption due to the wireless communication protocol will be ignored in this paper, it is not the focus of the presented work. Nevertheless, depending on the data frames nature and the implementation of processForecastOutput(*o*) function, the whole algorithm can be used for different applications. In the present work, the goal is to predict the indoor temperature, and thus every data frame is a temperature value given by a wireless node.


**Algorithm 1** Main procedure for sink node.
1:initializeWSN()2:initializeWeights()3:**while true do**4: *v* = waitForDataFrame()5: *t* = askForCurrentTimeStamp()6: *o* = processSampleOnLine(*v*, *t*)7: processForecastOutput(*o*)8:**end while**


### 4.2. On-Line Sample Processing

The subroutine processSampleOnLine(*v*, *t*) is displayed at Algorithm 2. This subroutine receives a pair of temperature frame *v* and the timestamp *t* in seconds, and executes one iteration of the on-line algorithm. This algorithm is responsible for the computation of mean values of temperature every *Q* seconds. Once a mean value is available (which happens every *Q* seconds), the algorithm calls the procedure TrainAndForecast(*v_q_*) which computes the first order differentiation of these means and adjusts the model weights. At the presented work, *Q* = 900 s (15 min, a quarter) because it is a reasonable value for temperatures.

**Figure 4:**
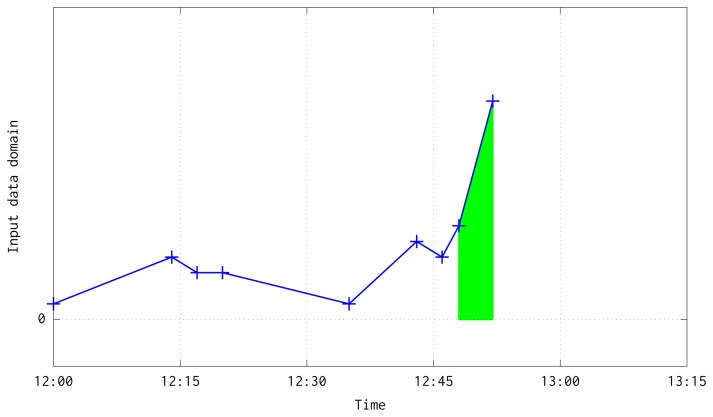
Illustration about the integration process of previous and current input data pairs.

During temperature mean computation, due to the non deterministic nature of the WSN, the data frames maybe are not equidistant in time. This algorithm considers this issue implementing an procedure of aggregation for the mean temperature computation (For this aggregation, it is assumed that every node is sensing similar temperatures, so their readings can be mixed-up without problems.). Every pair of consecutive input data, which belongs to the same time quarter, are integrated and aggregated to an accumulator variable (see line 18 at Algorithm 2) following this equation:

(14) $$\text{A}\left(t_{i},v_{i},t_{i+1},v_{i+1}\right)=\frac{t_{i+1}-t_{i}}{Q}\cdot \frac{1}{2}\left(v_{i}+v_{i+1}\right)$$

where 〈*t_i_*, *v_i_*〉 and 〈*t_i_*_+1_, *v_i_*_+1_〉 are the pair of input data. The Figure 4 shows a graphical illustration of this process for the case when the pair of data are into the same quarter.


**Algorithm 2**
processSampleOnLine(*v*,*t*)
**Require:**
*v*, *t* are *real numbers* with data value and timestamp in seconds. *v′*, *t′*, 
$q_{t}^{\prime }$, *v_q_* are *static variables* initialized with invalid values, and used by the algorithm to store the data value, the time and quarter number in previous function call, and in the end to aggregate the data value for current time quarter (the algorithm computes the mean every quarter.) The *constant Q* = 900 is the number of seconds in a quarter. The algorithm interpolates quarter mean values when they are missing, up to a maximum of *M* = 4 quarters.**Ensure:** A prediction vector, in case it is possible to be computed, otherwise it returns a NULL value.1:
$q_{t}=\left\lfloor \frac{t}{Q}\right\rfloor$2:**if**
*t′* is not a valid value **then**3: 
$v_{q}\leftarrow v\cdot \frac{\left(t\mathrm{mod}Q\right)}{Q}$// Initializes accumulated data for aggregation4:**else**5: **if**
$q_{t}-q_{t}^{\prime }>M$
**then** // Quarter change limit exceeded6:  RESET()7: **else**8:  〈*m*, *b*〉 = 

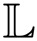
(*t*′, *v*′, *t, v*) // Interpolates line *v_i_* = *m* · *t_i_* + *b* using Equation (15)9:  
$t_{i}=Q\cdot \left(q_{t}^{\prime }+1\right)$10:  **while**
*t_i_* ≤ *t*
**do**11:   *v_ti_* = *t_i_* · *m*+*b*12:   *v_q_* ← *v_q_* + 

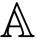
(*t′*, *v′*, *t_i_*, *v_t_i__*) // Aggregates data change following Equation (14)13:   *o_ti_* = trainAndForecast(*v_q_*) // Last o*_t_i__* is stored to be returned at function end14:   *v_q_* ← 0.0; *v′* ← *v_t_i__*; *t′* ← *t_i_*15:   *t_i_* ← *t_i_* + *Q*16:  **end while**17:  **if**
*t′*<*t*
**then**18:   *v_q_* ← *v_q_* + 

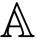
(*t′*, *v′*, *t*, *v*) // Aggregates last data change following Equation (14)19:  **end if**20: **end if**21:**end if**22:*t′* ← *t*; *v′* ← *v*; 
$q_{t}^{\prime }\leftarrow q_{t}$23:return o*_t_i__* if available, otherwise NULL


**Figure 5:**
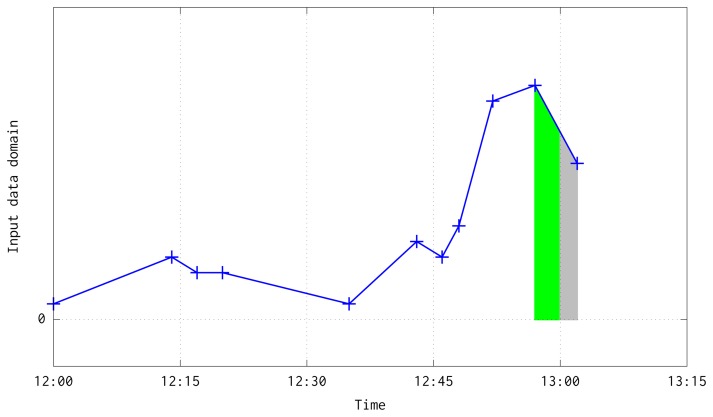
Illustration about the integration process of previous and current input data pairs when both are in different but in consecutive quarters. In green color has been shown the interpolation computed until 13:00, ending a quarter mean value computation. In gray color has been depicted the interpolation that would be aggregated to the quarter ending at 13:15.

**Figure 6:**
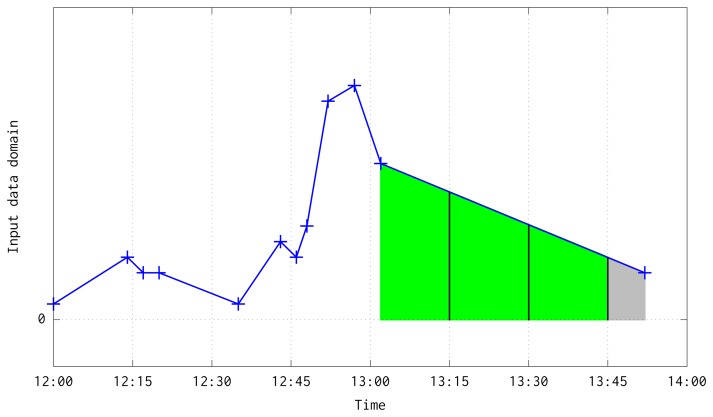
Illustration about the interpolation and integration of previous and current input data pairs when they are in non consecutive quarters. In green color has been depicted the interpolation of the computed quarters that end at 13:15, 13:30 and 13:45. In gray color has been shown the interpolation aggregated to the following quarter that would end at 14:00.

This aggregation has two boundary cases: when two consecutive pairs are in a different but consecutive time quarters (see Figure 5); or when two consecutive pairs are in different and non consecutive quarters due to the lost of a large number of frames (see Figure 6). In both situations, besides the case when a quarter is fully processed, are solved at the loop at line 10. Before the loop, it is interpolated a line equation which follows the temperature slope between the pair of available input data, computed with the next slope-intercept linear equation:

(15) $$\text{L}\left(t_{i},v_{i},t_{i+1},v_{i+1}\right)=\langle \begin{array}{cc} m=\frac{v_{i+1}-v_{i}}{t_{i+1}-v_{i}}, & b=v_{i}-m\cdot t_{i} \end{array}\rangle$$

The loop begins at the start point, and traverses the line segment by *Q* seconds increments, computing the mean temperature of every possible quarter between both input pairs. When a quarter is completed, its mean value is given to the subroutine trainAndForecast(*v_q_*). In the extreme case of losing a huge number of frames, the whole system is reset at line 6 of Algorithm 2, starting the process again but without initializing the model weights (This reset procedure also initializes the static variables of Algorithm 3).

The memory requirement for subroutine processSampleOnLine(*v*, *t*) is again negligible. It uses only a few static variables to aggregate the data values and/or interpolate the lost quarter values. For temperature, data values are in °C.

### 4.3. On-Line Training and Forecast using Back-Propagation

The last subroutine is depicted at Algorithm 3. It computes the difference between two consecutive quarter means, and stores them into an auxiliary circular buffer *B* with length *p* + *q*. A counter *k* is used to control the number of items in the buffer, controlling if it is possible to produce a forecast, and/or when it is feasible to perform one training step of the model. In particular, the forecast condition is true when the buffer counter *k* is greater or equal to the model's input size *p*. On the other hand, the train condition is true, when the buffer counter *k* is greater or equal to the sum of model's input *p* and output *q* sizes.

The algorithm uses a static variable *v′* where the value given at previous function call is stored. Thus, in the first call, the if statement at line 1 is not executed. In the following calls, the first order differentiation is computed at line 2 and the counter of elements is increased by one unit. The training condition is checked at line 4, and in case of success the input/output mapping is taken from the circular buffer *B* and the model is updated following BP Equations (B1)–(B8). The forecast condition is checked at line 13, and in case of success the input is taken from the last *p* items of the buffer *B*, then the forecast is produced following Equations (B1)–(B3). Finally, the output vector is dedifferentiated by computing the cumulative sum of the model output and adding it up with all the vector components and the input data value of current quarter.

Note that the reset call at line 6 of Algorithm 2 also initializes the static variables of this one. This algorithm has more critical memory requirements, due to the circular buffer *B*. The length of such buffer is *p* + *q*, and in the experimentation these values are *p* = *q* = 8, therefore, this algorithm needs 16 real numbers, that using 32 bit real numbers, corresponds to 64 bytes. Thus the total memory consumption needed by the whole algorithm (BP + on-line control) in the worst case adds up to 64 + 736 = 800 bytes.


**Algorithm 3**
trainAndForecast(*v_q_*)
**Require:**
*v_q_* is a *real number* with the value at current quarter. *v′* and *k* = 0 are *static variables*, the first one stores a quarter value given in previous function call and it is initialized with an invalid value. The second one is a counter initialized with 0 needed to access the auxiliary buffer *B. p*, *q* are *constants* with the input size, output size and buffer size respectively. *B* is a *static circular buffer* with *p* + *q* length. For simplicity, *B* is indexed with any integer value *i* ≥ 0, assuming that *i* mod (*p* + *q*) is needed to access to valid positions. *B* buffer stores the value difference between two time quarters. The forecast starts when the counter *k* is *p*, and the training starts when the counter *k* is at least *p* + *q*. The weight matrices **W***_j_* and **b***_j_* (both randomly initialized at start), activation vectors **h***_j_* and error gradients **δ***_j_* are *global variables*, used by forward, backprop and update functions. η_0_ is the initial learning rate, γ is the learning rate decay value, and ϵ is the weight decay, all of them are *constants*. These last three parameters have been set after a grid search optimization is done, with different values depending on the model. *α* = 0 is another *static variable* which is the number of performed learning iterations.**Ensure:** The algorithm trains the model using functions forward, backprop and update, and returns the forecast at current time quarter, or NULL if it cannot be computed.1:**if**
$v_{q}^{\prime }$ is a valid value **then**2: 
$B\left[k\right]=v_{q}-v_{q}^{\prime }$3: *k* ← *k* + 14: **if**
*k* >= *p* + *q*
**then** // Train when buffer *B* is full5:  **x** = *B*[(*k* − *p* − *q*) : (*k* − *q* − 1)]6:  **y** = *B*[(*k* − *q*) : (*k* − 1)]7:  **ŷ** = forward (**x**) // Following Equations (B1)–(B3)8:  backprop(**ŷ**, **y**) // Following Equations (B4)–(B6)9:  
$\eta =\frac{\eta_{0}}{{\left(1+\alpha \cdot \eta_{0}\right)}^{\gamma }}$10:  update(η, ϵ) // Following Equations (B7)–(B8)11:  *α* ← *α* + 112: **end if**13: **if**
*k* >= *p* then // Compute forecast14:  **x** = *B*[(*k* − *p*) : (*k* − 1)]15:  **ŷ** = forward (**x**) // Following Equations (B1)–(B3)16:  **o** = cumSum(**ŷ**) + *v_q_* // The outputs vector **ŷ** is dedifferentiated by computing the cumulative sum of the vector and adding-up current quarter value to all vector components17: **end if**18:**end if**19:
$v_{q}^{\prime }\leftarrow v_{q}$20:**return** o if available, otherwise NULL


In this algorithm the training and forecasting procedures are delayed by *q* iterations (*q* time quarters). Thus the algorithm produces forecasts using a model trained with data of *q* quarters in the past.

### 4.4. Last Remarks

**Figure 7:**
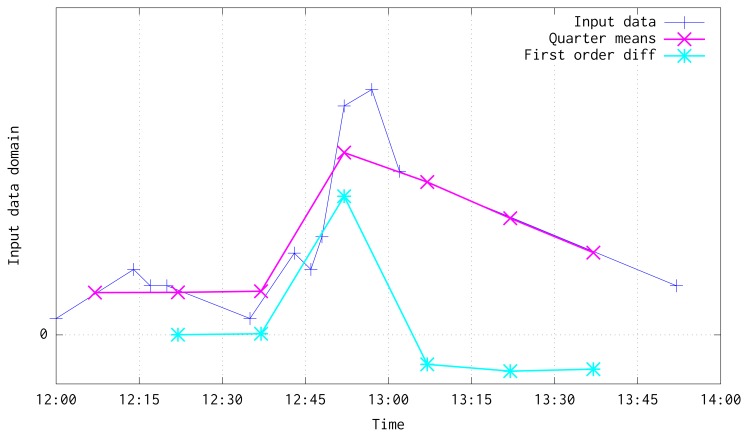
Illustration of input data, means and first order differentiated result.

As has been stated before, following an on-line method, this algorithm trains an ANN model for time series forecasting. In order to improve the model performance, the input data is aggregated and filtered by computing its mean every *Q* seconds. After that, first order differentiation of the data is computed, and the model is trained to predict the differentiated series. Figure 7 shows an illustration of input data, its transformation into means every *Q* seconds, and the differentiated series used to train the system. Mean and differentiated series have been plotted in the mid-point of every *Q* window.

This algorithm has been proposed to receive as input a fixed number of delayed past values. This limits the model ability to learn and forecast variables which are conditioned to exogenous data (For instance, in case of indoor air temperature prediction, HVAC operations and other related variables should be taken into account to ensure good model performance). Nevertheless, it is straightforward to extend the algorithm to receive additional data as input, which would be passed directly to the model as covariates.

## 5. Results and Discussion

For the study of the proposed algorithm, two case studies have been completed. The first one is a simulation using artificially generated data, with a very large number of iterations in order to have a baseline to compare model behavior and accuracy estimate. The second one is a real application for indoor temperature forecasting, using the dataset SML2010 DataSet [22] at UCI Machine Learning repository [23]. The SML2010 DataSet has been created by the ESAI research team (the authors of the present paper) at the Universidad CEU Cardenal Herrera, monitoring the data captured from a solar domotic house that participated in the Solar Decathlon Europe 2010, a world competition of energy efficiency. Such a dataset has been employed as it is tidy, *i.e.*, it has been cleaned and structured, and it is ready for analysis.

### 5.1. A Simulation Study

**Figure 8:**
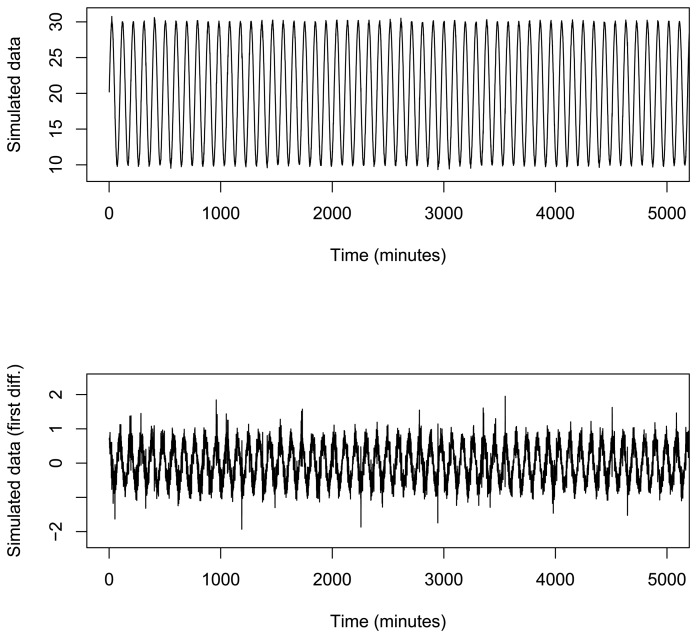
First 5000 simulated time series data and first differences.

A simulation study has been carried out to deeply explore the algorithm behavior. The simulated dataset contains 10^6^ data, based on a sinus function in different time points along 8500 h, with centigrade degree values between 10 and 30 (more variability than real temperature values). Furthermore, the distance between two consecutive values was randomly taken from a range of [20, 40] s. Sin values have also been randomly modified with noise in a range of [−1.5, 1.5]. The original dataset was on-line preprocessed by the mean in order to obtain one value of temperature every 15 minutes. Moreover, in order to increase model generalization, first differences on preprocessed data were calculated, obtaining the final dataset that was modeled. Note that the proposed on-line BP algorithm computes the mean and first order differences on-the-fly during the training process. This dataset is shown in Figure 8.

**Figure 9:**
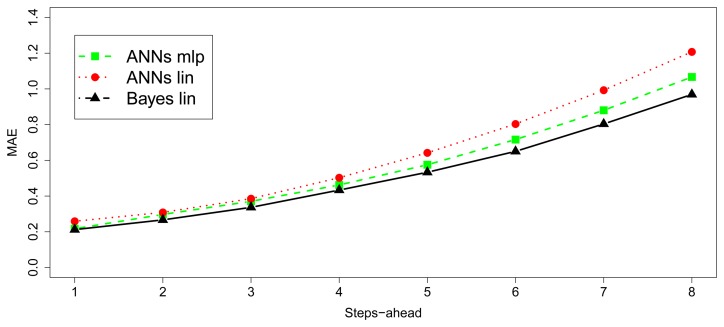
MAE of each step-ahead for last 15,000 for sin data.

**Figure 10:**
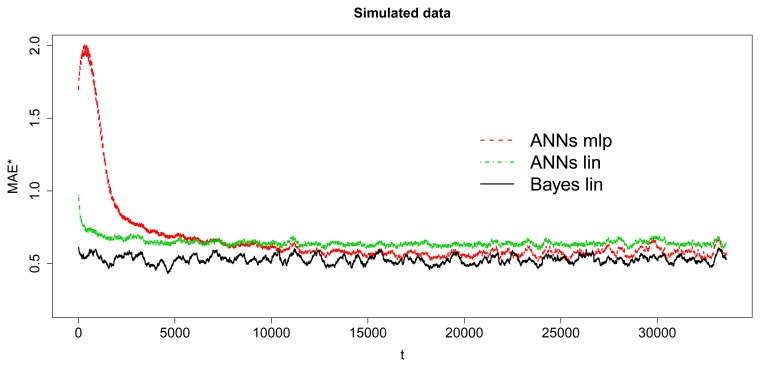
Smoothed MAE^★^—sin data.

Figures 9 and 10 show the MAE and MAE^★^ behavior. First, the Figure 9 illustrates how the errors evolve with the number of steps-ahead. The first 15,000 observations have not been considered in this calculation, because they belong to the period in which the convergence of the algorithms had not been reached. Second, the Figure 10 shows the smoothed MAE^★^ behavior, calculated by 10 values window length to avoid randomness noise over the time and the Table 1 depicts the MAE summarized obtained from the dataset for the baseline Bayesian model and the implementable ANN models. The input/output structure was defined choosing 8 values as input and 8 future values as output. Thus, the model receives at each moment as input, the last eight values in time series and it must predict next eight values (next two hours by steps of 15 min).

Figure 10 and Table 1 demonstrate the comparison between the Bayesian baseline and the performance obtained by the linear and MLP models. It is possible to observe how the Bayesian baseline is able to obtain low errors almost from the first iteration, because of its better utilization of the available information. The structure employed in this baseline model is higher than the other two methods. The baseline model operates in each iteration with a *n* × *p* matrix **X**, with *n* = *p* + 1, and a vector *n* × 1 vector **Y**_._*_i_*. However, ANN models handles only a 1 × *p* vector **x** and a vector 1 × 1 vector **y**. Regarding ANN models it is observed that the linear model learns faster than MLP, nevertheless MLP achieves better error in the long-term. As expected, the more complex the model is the more difficult the learning process, but it achieves similar results to the Bayesian baseline in the long run.

### 5.2. Real Application: Temperature Forecasting in a Solar House

**Figure 11:**
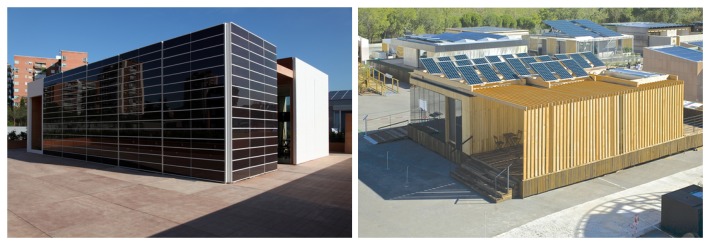
Solar-powered houses: SMLhouse (**left**) and SMLsystem (**right**).

As was mentioned before, the School of Technical Sciences at the University CEU Cardenal Herrera (CEU-UCH) participated in 2010 and 2012 at Solar Decathlon Europe competition building two solar-powered houses known as SMLhouse and SMLSystem respectively (Figure 11). One of the technologies integrated in that houses was a monitoring system developed to collect all the data related with energy consumption and other variables as indoor/outdoor temperature, CO2, *etc.* A tidy database was created and it has been exploited to develop different ANN models for prediction purposes in previous research projects.

**Figure 12:**
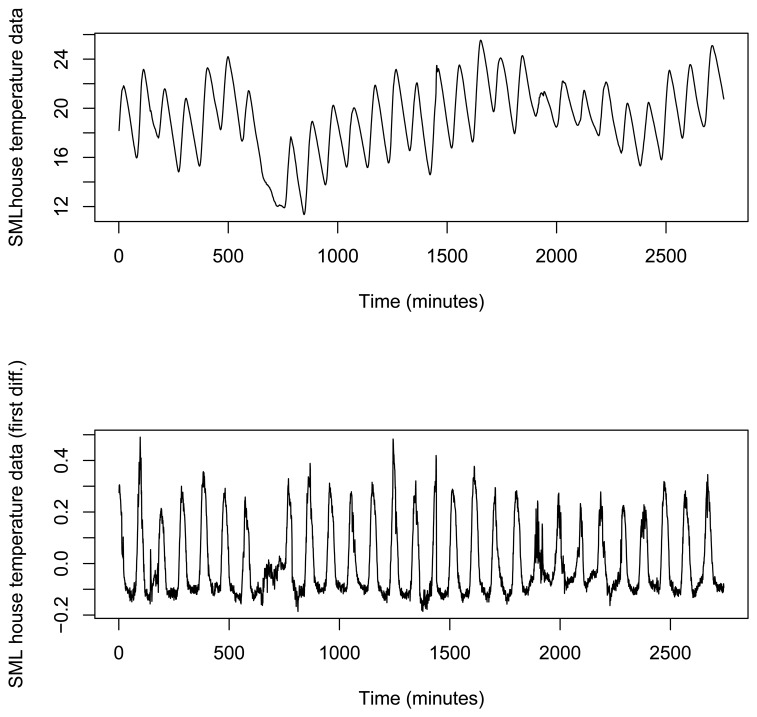
SMLhouse time series data.

As stated before, a tidy dataset, with 673 h (28 days) of real temperature data, recorded every 15 min is available (2688 equally spaced temperature data) and it has been utilized to study the performance of the overall system. This dataset and its first difference is shown in Figure 12.

**Figure 13:**
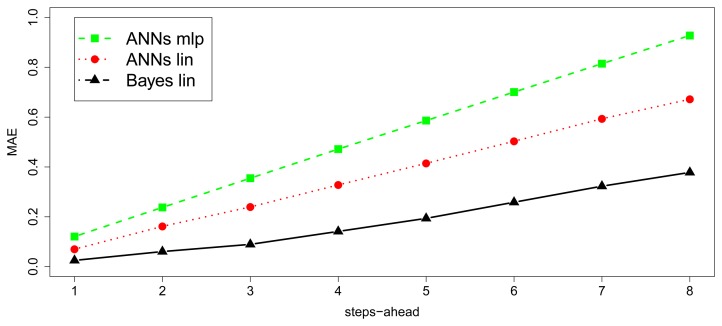
MAE of each step-ahead for SML house forecasts.

**Figure 14:**
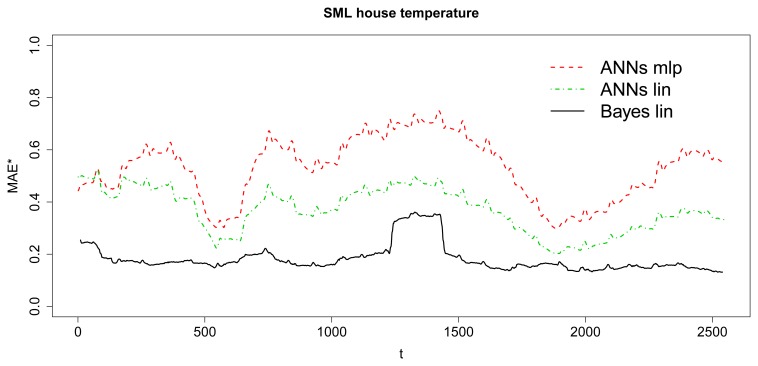
Smoothed MAE ^★^—SML house.

In the same way as the study of the simulated data, Figures 13 and 14 show de MAE and MAE^★^ behavior. Figure 13 illustrates how the errors evolve with the number of steps-ahead and Figure 14 visualizes the smoothed MAE^★^ behavior, calculated by 10 values window length to avoid randomness noise over the time. Table 2 shows the summarized MAE obtained with the dataset for the baseline Bayesian model and the implementable ANN models. The input/output structure was defined choosing 8 values as input and 8 future values as output. Furthermore, the model receives at each moment as input the last eight values in time series and must predict the next eight ones (next two hours by step of 15 min), as it has been described for the baseline before. Because of the short observation period, compared to the observation period of simulated data, ANNs models do not converge to the baseline model results. However, as it has been demonstrated in the simulation study, it is expected that as the time evolves, the errors tend to be equal. Anyway, although it can be observed how the Bayesian baseline is able to obtain low errors again, nevertheless the absolute differences between this method and ANN models is negligible in practical terms.

The experimental results obtained seem very promising, as we were able to implement a complex algorithm in a simple hardware device to predict time series accurately. In previous research, published in the journal Energy and Buildings [24] this kind of algorithms, in analogous situations but with additional variables, were able to obtain superior predictions and converge to low errors in lower periods of time. Such issue makes us to consider dealing with the possibility of using the present algorithm and the same ideas to develop a new one that will employ similar variables, as the previous work, but to be implemented in hardware devices with higher resources, keeping the restrictions of using cheap and small-sized microcontrollers.

## 6. Conclusions

This paper describes how to implement an artificial neural network in a low cost system-on-chip to develop an autonomous intelligent wireless sensor network. The model is able to produce predictions of temperature coming from four sensor nodes based on an on-line learning approach. The idea behind this project is to evaluate if it is possible to develop, in a system with very few resources as the MCU 8051, the necessary source code to integrate a neural network that learns a time series in an on-line strategy, *i.e.*, without any historical database. It is obvious that all kind of problems cannot be afforded with this technology/approach. Nevertheless, on some physical measurements, represented as a stationary time series, it is possible to apply an on-line learning paradigm. And it makes attainable to generate predictions of time series in feasible temporal windows, in the present case study in few days. That means that it is conceivable to place a low cost and small intelligent system in a totally unknown environment to learn its dynamics very rapid and with a wireless technology.

The on-line learning approach is suboptimal in terms of the model accuracy. The proposed algorithm is able to learn accurate forecast models, but as stated at Section 3.2, learning in a sequential way could harm the model learning. Stochastic gradient descent algorithms are based on random sampling of training dataset, and minor changes in the proposed algorithm can be performed to allow some class of random sampling in the real-time data stream. The implementation of these ideas in more complex devices would allow to increase model and of course algorithm complexity, in time and space, improving the experimental results shown in this work. An idea for the future is to scale this project to architectures more complex but starting with an efficient algorithm as a baseline.

Finally, some issues would arise when using the proposed models to predict the temperature of a room where HVAC system is operating. Air temperature would be affected by HVAC operations, among other exogenous variables, thus forecasting only with a fixed number of past delayed values would be not enough. However, this problem can be tackled by extending the model with additional input information regarding the operations performed by the HVAC system and other exogenous variables that determine the room temperature.

## Figures and Tables

**Table 1:** Comparison between Bayesian baseline and ANNs models. Both methods has a *p* = 8 inputs and *q* = 8 outputs. The MLP has a hidden layer with *h* = 8 neurons.

**Method**	**Min.**	**Q1**	**Q2**	**Mean**	**Q3**	**Max.**
Baseline (Bayesian standard)	0.047	0.248	0.442	0.528	0.720	4.227
Lin	0.036	0.368	0.632	0.648	0.877	2.991
MLP	0.046	0.323	0.553	0.662	0.871	3.708

**Table 2:** Comparison between Bayesian baseline and ANNs models. Both methods has a *p* = 8 inputs and *q* = 8 outputs.

**Method**	**Min.**	**Q1**	**Q2**	**Mean**	**Q3**	**Max.**
Baseline (Bayesian standard)	0.008	0.087	0.141	0.184	0.222	4.295
Lin ANN	0.011	0.213	0.309	0.373	0.432	1.978
MLP ANN	0.013	0.233	0.466	0.527	0.734	2.109

## Appendix

### A. Standard Bayesian Linear Model Estimation

This section describes some aspects of the standard bayesian estimation for regression linear model parameters in which our work is based.

#### A.1. Non-Informative Prior Distributions

The linear model could be written:

(A1) $$\text{y}|\text{w},e^{2},\text{X}∼N\left(\text{Xw},e^{2}\text{I}\right)$$

The non-informative prior distribution most commonly used for linear regression is:

(A2) $$P\left(\text{w}|e^{2}\right)\propto \frac{1}{e^{2}}$$

The posterior distribution of **w** given *e*^2^ and the marginal posterior distribution of *e*^2^ could be obtained analytically as follows:

(A3) $$\text{w}|e^{2},Y∼\text{Normal}\left(\text{w},V_{\text{w}}e^{2}\right)$$

(A4) $$e^{2}|Y∼\text{Inverse}\chi^{2}\left(n-p,s^{2}\right)$$

The parameters estimation of these distributions could be calculated as follows:

(A5) $$\hat{\text{w}}={\left(X^{\text{⊤}}X\right)}^{-1}X^{\text{⊤}}\text{y}$$

(A6) $${\text{V}}_{\text{w}}={\left(X^{\text{⊤}}X\right)}^{-1}$$

(A7) $$s^{2}=\frac{{\left(\text{y}-X\hat{\text{w}}\right)}^{\text{⊤}}\left(y-X\hat{\text{w}}\right)}{n-p}$$

The symbol ⊤ denotes matrix transposition. It would be needed *n* > *p* for carry out parameters estimation and *n* = *p* + 1 has been considered in the process estimation (*p* × (*p* + 1) data).

If *q* must be predicted in each time step, *q* linear models must be estimated. Thus, *q* estimations of vector **w** (**w***_i_* for *i* = 1, …*q*) could be represented as **W** matrix, that contains in each of its *q* columns the estimated parameter vector **w***_i_* for each prediction.

$${\text{W}}_{.i}=w_{i}$$

In the same way, vector of predictions **y** becomes in a matrix **Y** with *q* columns. Vector column **W**_._*_i_* corresponds with parameter vector in the linear model with response vector **Y**_._*_i_*. Each of these models has the same predictor matrix **X**.

At time *t* = *p* + *p*+1 a first estimation **W**[0] is available and can be used to make predictions at time *t* + 1. The predictive distribution, **Ŷ**, given a new set of predictors **X***_p_* has mean:

(A8) $$\begin{array}{cc} {\hat{Y}}_{.i}={\text{X}}_{p}\hat{W}{\left[0\right]}_{.i} & i=1,\ldots ,q \end{array}$$

With variance for this estimation:

(A9) $$\text{var}\left({\hat{Y}}_{.i}|{\text{e}}_{.i}^{2},Y\right)=\left(I+{\text{X}}_{p}{\text{V}}_{w}{\text{X}}_{p}^{т}\right){\text{e}}_{.i}^{2}$$

It is necessary to solve matrix products and inverse matrices with dimension (*p* × *p*).

#### A.2. Informative Prior Distribution

Furthermore, in the following time points *t*, if last parameter estimation is **Ŵ**[*t* − 1], *s*^2^[*t* − 1] and *V***_w_**[*t* − 1] and we have new data **X**[*t*] and **Y**[*t*], new parameters estimation at this point of time can be computed by treating the prior as additional data points, and then weighting their contribution to the new estimation [20]. To perform the computations, for each prediction value **Y**[*t*]_._*_i_* (*i* = 1, …, *q*)it is necessary to construct a new vector of observations ${\text{Y}}_{.i}^{*}$ with new data and last parameter estimations, and predictor matrix **X***, and weight matrix **Σ** based on previous variance parameters estimation as follows:

(A10) $${\text{Y}}_{.i}^{*}=\left[\begin{array}{c} \text{Y}{\left[t\right]}_{.i}\\ \hat{\text{W}}{\left[t-1\right]}_{.i} \end{array}\right]$$

(A11) $${\text{X}}^{*}=\left[\begin{array}{c} \text{X}\left[t\right]\\ {\text{I}}_{p} \end{array}\right]$$

(A12) $$\Sigma =\left[\begin{array}{cc} {\text{I}}_{n} & 0\\ 0 & {\text{V}}_{w}\left[t-1\right]{\text{s}}_{i}^{2}\left[t-1\right] \end{array}\right]$$

New parameters estimation at time t could be written as:

(A13) $$\begin{array}{cc} \hat{\text{W}}{\left[t\right]}_{.i}={\left({\text{X}}^{*\text{⊤}}\Sigma^{-1}{\text{X}}^{*}\right)}^{-1}{\text{X}}^{*\text{⊤}}\Sigma^{-1}{\text{Y}}_{.i}^{*} & i=1,\ldots ,q \end{array}$$

(A14) $${\text{V}}_{\text{w}}\left[t\right]={\left({\text{X}}^{*\text{⊤}}\Sigma^{-1}{\text{X}}^{*}\right)}^{-1}$$

(A15) $${\text{s}}_{i}^{2}\left[t\right]=\frac{n_{0}{\text{s}}_{0i}^{2}+n_{1}{\text{s}}_{1i}^{2}}{n_{0}+n_{1}}$$

where

(A16) $${\text{s}}_{1i}^{2}=\frac{{\left(\text{Y}{\left[t\right]}_{.i}-\text{X}\left[t\right]\hat{\text{W}}{\left[t\right]}_{.i}\right)}^{\text{⊤}}\left(\text{Y}{\left[t\right]}_{.i}-\text{X}\left[t\right]\hat{\text{W}}{\left[t\right]}_{.i}\right)}{n_{1}-p}$$

moreover, $s_{0}^{2}$ is the variance estimation at time *t* − 1 and *n*_0_ is its degrees of freedom, and *n*_1_ is the degrees of freedom in the new data variance estimation. Computational cost is higher than first step, with more matricial products and more inverse matrices calculus. The Bayesian standard parameters estimation is a simple process but with high resources requirements.

### B. On-Line Back-Propagation Derivation for ANN Models

This section mathematically formalizes the BP algorithm, which is widely known in ANN related literature. These equations are described for completeness and to help the understanding of memory requirements stated at Section 4.

For any ANN model, with zero or more hidden layers, the procedure of computing its output **ŷ** given its inputs **x** is known as *forward* step. The following equations show the computation needed during forward step:

(B1) $${\text{h}}_{0}=\text{x}$$

(B2) $$\begin{array}{cc} {\text{h}}_{j}=s\left({\text{W}}_{j}\cdot {\text{h}}_{j-1}+{\text{b}}_{j}\right), & \text{for}1\leq j \end{array}<N$$

(B3) $$\hat{\text{y}}={\text{W}}_{N}\cdot {\text{h}}_{N-1}+{\text{b}}_{N}$$

being $s\left(z\right)=\frac{1}{1+\exp\left(-z\right)}$ the logistic function and *N* the number of layers in the network, that is, the number of hidden layers plus one because of the output layer. With forward step, all hidden **h***_j_* and output layer **ŷ** activations are computed. Following the forward step, it is possible to compute the loss *L*(**ŷ**, **y**) of the ANN output respect to the given desired output y by using the mean square error. The derivation of this loss function respect to every output and hidden layer is computed by the *backprop* step by means of the next equations:

(B4) $$L\left(\hat{\text{y}},\text{y}\right)=\frac{1}{2}{\left\|\hat{\text{y}}-\text{y}\right\|}_{2}^{2}+\frac{\epsilon }{2}\sum_{j=1}^{N}\sum_{\omega \in {\text{W}}_{j}}\omega^{2}$$

(B5) $$\delta_{N}=\frac{\partial L\left(\text{x},\text{y}\right)}{\partial \hat{y}}=\hat{y}-\text{y}$$

(B6) $$\begin{array}{cc} \delta_{j}=\frac{\partial L\left(\text{x},\text{y}\right)}{\partial {\text{h}}_{j}}={\text{h}}_{j}^{\prime }\circ \left({\text{W}}_{j+1}^{\text{⊤}}\cdot \delta_{j+1}\right) & \text{for}1\leq j<N \end{array}$$

being **A** ∘ **B** the component-wise product between two vectors and ${\text{h}}_{j}^{\prime }$ the derivative of the logistic function, that corresponds to ${\text{h}}_{j}^{\prime }\circ \left(1-{\text{h}}_{j}^{\prime }\right)$. After the forward and backprop steps, all activations and hidden and output layer error gradients **δ***_j_* are available. Thus, the gradient of the loss function respect to the weight matrices and bias vectors can be computed. Following this gradient, all weights and biases are updated. Such phase is denoted by *update* step, and its equations are:

(B7) $$\begin{array}{cc} {\text{W}}_{j}^{\left(e+1\right)}={\text{W}}_{j}^{\left(e\right)}-\eta \frac{\partial L\left(\text{x},\text{y}\right)}{\partial {\text{W}}_{j}^{\left(e\right)}}={\text{W}}_{j}^{\left(e\right)}-\eta \cdot \left(\delta_{j}⊗{\text{h}}_{j-1}+\epsilon {\text{W}}_{j}^{\left(e\right)}\right) & \text{for} \end{array}1\leq j\leq N$$

(B8) $$\begin{array}{cc} {\text{b}}_{j}^{\left(e+1\right)}={\text{b}}_{j}^{\left(e\right)}-\eta \frac{\partial L\left(\text{x},\text{y}\right)}{\partial {\text{b}}_{j}^{\left(e\right)}}={\text{b}}_{j}^{\left(e\right)}-\eta \cdot \delta_{j}, & \text{for}1\leq j\leq N \end{array}$$

being **A** ⨂ **B** the outer product between two vectors.

## References

[1] Zheng J., Jamalipour A. (2009). Wireless Sensor Networks: A Networking Perspective.

[2] Ferreira P., Ruano A., Silva S., Conceição E. (2012). Neural networks based predictive control for thermal comfort and energy savings in public buildings. Energy Build..

[3] Álvarez J., Redondo J., Camponogara E., Normey-Rico J., Berenguel M., Ortigosa P. (2012). Optimizing building comfort temperature regulation via model predictive control. Energy Build..

[4] Wu C.L., Chau K.W., Li Y.S. (2009). Predicting monthly streamflow using data-driven models coupled with data-preprocessing techniques. Water Resour. Res..

[5] Taormina R., wing Chau K., Sethi R. (2012). Artificial neural network simulation of hourly groundwater levels in a coastal aquifer system of the Venice lagoon. Eng. Appl. AI.

[6] Karatasou S., Santamouris M., Geros V. (2006). Modeling and predicting building's energy use with artificial neural networks: Methods and results. Energy Build..

[7] Ruano A., Crispim E., Conceição E., Lúcio M. (2006). Prediction of building's temperature using neural networks models. Energy Build..

[8] Zamora-Martínez F., Romeu P., Botella-Rocamora P., Pardo J. (2013). Towards Energy Efficiency: Forecasting Indoor Temperature via Multivariate Analysis. Energies.

[9] Cho S., Zaheer-uddin M. (2003). Predictive control of intermittently operated radiant floor heating systems. Energy Convers. Manag..

[10] Frandina S., Gori M., Lippi M., Maggini M., Melacci S. (2013). Variational Foundations of Online Backpropagation. Artificial Neural Networks and Machine Learning—ICANN 2013.

[11] Calvet S., Campelo J.C., Estellés F., Perles A., Mercado R., Serrano J.J. (2014). Suitability Evaluation of Multipoint Simultaneous CO_2_ Sampling Wireless Sensors for Livestock Buildings. Sensors.

[12] Dargie W., Poellabauer C. (2010). Fundamentals of Wireless Sensor Networks: Theory and Practice.

[13] Sohraby K., Minoli D., Znati T. (2007). Wireless Sensor Networks: Technology, Protocols, and Applications.

[14] Texas Instruments CC1110F32. Low-Power SoC (System-on-Chip) with MCU, Memory, Sub-1 GHz RF Transceiver, and USB Controller. http://www.ti.com/product/cc1110f32.

[15] Chatfield C. (2013). The Analysis of Time Series: An Introduction.

[16] Anderson T.W. (2011). The Statistical Analysis of Time Series.

[17] Montgomery D.C., Jennings C.L., Kulahci M. (2011). Introduction to Time Series Analysis and Forecasting.

[18] Rumelhart D.E., Hinton G.E., Williams R.J. (1988). Neurocomputing: Foundations of Research.

[19] Brown M., Harris C. (1994). Neurofuzzy Adaptive Modelling and Control.

[20] Gelman A., Carlin J.B., Stern H.S., Dunson D.B., Vehtari A., Rubin D.B. (2013). Bayesian Data Analysis.

[21] Solar Decathlon Europe 2012 http://www.sdeurope.org/.

[22] Zamora-Martínez F., Romeu-Guallart P., Pardo J., SML2010 Data Set https://archive.ics.uci.edu/ml/datasets/SML2010.

[23] Bache K., Lichman M., UCI Machine Learning Repository http://archive.ics.uci.edu/ml/.

[24] Zamora-Martínez F., Romeu P., Botella-Rocamora P., Pardo J. (2014). On-line learning of indoor temperature forecasting models towards energy efficiency. Energy Build.

